# Cerebrospinal fluid levels of neopterin are elevated in delirium after hip fracture

**DOI:** 10.1186/s12974-016-0636-1

**Published:** 2016-06-29

**Authors:** Roanna J. Hall, Leiv Otto Watne, Ane-Victoria Idland, Johan Raeder, Frede Frihagen, Alasdair M. J. MacLullich, Anne Cathrine Staff, Torgeir Bruun Wyller, Durk Fekkes

**Affiliations:** Edinburgh Delirium Research Group, University of Edinburgh, Edinburgh, Scotland; Centre for Cognitive Ageing and Cognitive Epidemiology, University of Edinburgh, Edinburgh, Scotland; Oslo Delirium Research Group, Department of Geriatric Medicine, Oslo University Hospital, PB 4950 Nydalen, N-0424 Oslo, Norway; Institute of Basic Medical Sciences, University of Oslo, Oslo, Norway; Institute of Clinical Medicine, University of Oslo, Oslo, Norway; Department of Anesthesiology, Oslo University Hospital, Oslo, Norway; Department of Orthopedic Surgery, Oslo University Hospital, Oslo, Norway; Department of Obstetrics and Gynaecology, Oslo University Hospital, Oslo, Norway; Department of Anaesthesiology, Erasmus MC, University Medical Center Rotterdam, Rotterdam, The Netherlands

**Keywords:** Delirium, Dementia, CSF, Cellular immunity, Oxidative stress

## Abstract

**Background:**

The inflammatory cell product neopterin is elevated in serum before and during delirium. This suggests a role for disordered cell-mediated immunity or oxidative stress. Cerebrospinal fluid (CSF) neopterin levels reflect brain neopterin levels more closely than serum levels. Here we hypothesized that CSF neopterin levels would be higher in delirium.

**Methods:**

In this prospective cohort study, 139 elderly patients with acute hip fracture were recruited in Oslo and Edinburgh. Delirium was diagnosed with the confusion assessment method performed daily pre-operatively and on the first 5 days post-operatively. Paired CSF and blood samples were collected at the onset of spinal anaesthesia. Neopterin levels were measured using high-performance liquid chromatography.

**Results:**

Sixty-four (46 %) of 139 hip fracture patients developed delirium perioperatively. CSF neopterin levels were higher in delirium compared to controls (median 29.6 vs 24.7 nmol/mL, *p* = 0.003), with highest levels in patients who developed delirium post-operatively. Serum neopterin levels were also higher in delirium (median 37.0 vs 27.1 nmol/mL, *p* = 0.003). CSF neopterin remained significantly associated with delirium after controlling for relevant risk factors. Higher neopterin levels were associated with poorer outcomes (death or new institutionalization) 1 year after surgery (*p* = 0.02 for CSF and *p* = 0.03 for serum).

**Conclusions:**

This study is the first to examine neopterin in CSF from patients with delirium. Our findings suggest potential roles for activation of cell-mediated immune responses or oxidative stress in the delirium process. High levels of serum or CSF neopterin in hip fracture patients may also be useful in predicting poor outcomes.

## Background

Delirium is a serious acute neuropsychiatric syndrome comprising acute and fluctuating deterioration in attention, cognition, perception, and level of consciousness [1]. Although delirium is extremely common [2], distressing [3] and associated with multiple adverse outcomes [4], the pathophysiology of delirium is still poorly understood [5] and there are no specific treatments.

There has been much speculation that delirium may involve inflammation. This is because infection is a frequent precipitant of delirium and human studies have found elevated pro-inflammatory cytokines in serum [6, 7] and cerebrospinal fluid (CSF) [6, 8] in patients with delirium. Animal studies further support a role for inflammation: in the presence of pre-existing neurodegenerative disease, moderate peripheral inflammatory stimulus induces a delirium-like transient behavioural change in conjunction with an exaggerated central inflammatory response [9, 10]. Though multiple mechanisms may be implicated, there is some evidence for a role of disordered cell-mediated immunity in delirium. Interferon-γ (IFN-γ) is important in stimulating the cell-mediated response, and higher baseline serum IFN-γ levels are associated with more severe delirium [11] and higher 6-month mortality [12]. There may also be a role for disordered oxidative stress in delirium. With its high metabolic demands, and poor antioxidant capabilities, the brain is highly vulnerable to damage from oxidative stress, and this could plausibly result in neuronal dysfunction under certain conditions [13]. Cardiopulmonary bypass surgery is a high-risk state for oxidative stress, and these pathways have been examined in post-operative delirium [13]. Karlidag et al. demonstrated that low pre-operative levels of the antioxidant enzyme catalase in blood predisposed to post-operative delirium after cardiac surgery [13]. Delirium in ICU patients is also associated with impaired oxidative metabolism [14].

The pteridine neopterin [15] was first isolated in 1963. In humans, neopterin is synthesized by monocytes, macrophages, microglia, and dendritic cells from guanosine triphosphate (GTP) in response to IFN-γ from activated CD4+ and CD8+ T cells and natural killer cells [15, 16]. Recently, neopterin has been shown to have a role in oxidative stress. It amplifies the cytotoxicity of reactive oxygen species from the macrophage oxidative burst, thereby increasing the cytocidal effects of the cell-mediated immune response [16]. Neopterin is being studied in many fields as a biomarker both for cell-mediated immunity and oxidative stress [16].

In the central nervous system (CNS), neopterin is produced by microglia [17]. CSF levels are elevated in CNS infections and in HIV, particularly HIV with concomitant neuropsychiatric sequelae [18]. Plasma levels of neopterin are elevated in Alzheimer’s dementia and correlate with cognitive decline [19]; however, levels in CSF were unchanged in one study comparing 20 patients with Alzheimer’s dementia with age- and sex-matched controls [20].

Neopterin levels have been little studied in delirium. Osse et al. have shown that high pre-operative plasma neopterin levels predict post-operative delirium following cardiac surgery, and high post-operative serum levels were also associated with delirium [21]. High plasma neopterin levels were associated with delirium in a study of elderly patients acutely admitted to a medical ward [22]. No studies have examined CSF neopterin levels in delirium.

This study tested the hypothesis that neopterin is elevated in CSF and in serum in delirium in acute hip fracture patients.

## Methods

### Participants

Hip fracture patients were recruited from Oslo University Hospital, Norway, between 2009 and 2012, and from the Royal Infirmary of Edinburgh, Scotland, between 2009 and 2011.

All patients recruited in Oslo were part of a randomized controlled trial evaluating the effect of an orthogeriatric model where hip fracture patients were treated in an acute geriatric ward [23]. There were no exclusion criteria related to place of living or age in the Oslo sample. However, patients were only included if the fracture was caused by a low energy trauma (defined as a fall from less than 1 metre). In Edinburgh, patients were excluded if they were under the age of 60, were nursing home residents, had significant Parkinson’s disease or had malignant or other comorbid disease such that prognosis was less than one year.

The patients were recruited in the emergency room by a member of the orthopaedic surgical team in Oslo and by a geriatrician (RH) in the Orthopaedic Trauma Unit in Edinburgh. Only patients who had both CSF and a pre-operative serum sample collected after recruitment were included in the present analysis.

### Assessments and procedures

Patients were screened for delirium once daily with the confusion assessment method (CAM) [24]. All patients were assessed pre-operatively and until day 5 post-operatively. Delirious patients were assessed until discharge. A geriatrician (LOW) and a trained research nurse performed the assessments in Oslo. In Edinburgh, one geriatrician (RH) did all the assessments.

In both hospitals, the CAM scores were based on information from nurses, close relatives and hospital records, in combination with a 10- to 30-min interview with the patient. Details of the delirium assessments were previously described [25]. Patients were regularly assessed on weekdays only, but staff members that had been working during weekends were interviewed every Monday and the case notes scrutinized for evidence of delirium. All sections of the notes were examined, and any comments on change in mental status were recorded. Delirium severity was assessed with the Memorial Delirium Assessment Scale (MDAS) [26] in Oslo and the Delirium Rating Scale Revised-98 (DRS-R98) [27] in Edinburgh. MDAS and DRS-R98 scores have a very high level of agreement, and to enable comparison, MDAS scores were generated retrospectively on patients in Edinburgh using a validated conversion rule [28].

The Informant Questionnaire on Cognitive Decline in the Elderly (IQCODE), 16 item version, was used as a measure of pre-fracture cognitive status [29]. The IQCODE was completed based on an interview with a close relative, staff in the nursing home or home nursing team, asking the informant to judge the responses based on patient’s status when last stable before the present fracture. A cutoff of 3.44 was used as an indicator of pre-fracture cognitive impairment [29]. In Oslo, the Barthel ADL Index [30] (0–20 scoring) was scored based on information from the same informants, emphasizing the person’s pre-fracture status. In Edinburgh, the Katz ADL [31] was used. Independence in ADL was defined as a Barthel ADL Index score of 19 or 20, or a Katz ADL score of 5 or 6. The Acute Physiology and Chronic Health Evaluation II (APACHE II) score on admission to hospital was calculated as a measure of physiological disturbance, though without information on arterial blood gases and haematocrit. Comorbid conditions were quantified with the Charlson Comorbidity Index [32].

### Sample collection, sample handling and laboratory procedures

Venous blood was collected in 5-mL serum tubes from hip fracture patients pre-operatively. After sampling, tubes were left in the vertical position for 30 min at room temperature for clotting before they were centrifuged. Aliquots of 200–500 μL were then stored at −80 °C in polypropylene tubes. CSF was collected at the onset of spinal anaesthesia before administration of the anaesthetic agent. CSF was collected in polypropylene tubes. The CSF was centrifuged within 4 h, and the supernatant was stored in aliquots of 100–1000 μL at −80 °C.

Samples were sent on dry ice to the University Medical Center Rotterdam for analyses. Neopterin in serum and CSF were measured by high-performance liquid chromatography (HPLC) after acid oxidation, as previously described [33]. The analyses were done in duplicate and the technician was blinded to clinical data. The quantitation was done by measuring peak heights and the recoveries were determined in every HPLC run by adding two different amounts of neopterin to serum (99–109 %) and/or CSF (97–101 %). In any subsequent HPLC run, six samples of the former run were re-analysed to check precision.

### Statistical analyses

Categorical variables were analysed by the Chi-square test. Continuous variables were analysed by Mann-Whitney tests or *t* tests depending on the distribution of the data. In our primary analysis, we compared the levels of neopterin in CSF and serum between patients with and without delirium by a Mann-Whitney *U* test. In a secondary analysis, we divided the patients into four subgroups based on delirium status and pre-fracture cognitive status (stratified by IQCODE, cut point 3.44):

1. Neither delirium nor chronic cognitive impairment
2. Chronic cognitive impairment, but no delirium
3. Delirium, but no chronic cognitive impairment
4. Delirium superimposed on chronic cognitive impairment

We also stratified the patients according to delirium status at the time of sampling:

1. Prevalent delirium—those who had delirium when CSF was taken
2. Incident delirium—those who were free from delirium when CSF was taken but developed delirium post-operatively
3. Subsyndromal delirium—those who at any point (pre-operatively or post-operatively) experienced some delirious symptoms (defined as at least two positive CAM features) but never fulfilled the criteria for full delirium [34]
4. Never delirium—those who never experienced delirium or subsyndromal delirium

The Kruskal-Wallis test was used to assess potential subgroup differences.

To assess the relationship between neopterin and delirium when adjusting for other covariates, we performed logistic regression analyses, one with serum neopterin and one with CSF neopterin as covariates, and both using ‘delirium at any time’ as the outcome variable. The inclusion of other covariates was based on a *p* value <0.1 in univariate analyses. All analyses were on pooled data from both centres. To assess the possibility that differences in diagnostic judgments between Oslo and Edinburgh could influence delirium rates, ‘centre’ was forced in as a covariate in both regression analyses.

Because a history of cancer and/or an ongoing infection and/or use of non-steroid anti-inflammatory drugs (NSAIDs)/acetylsalicyclic acid (ASA) might influence neopterin levels, we also ran analyses with patients with any of these excluded. We also assessed the association between neopterin and length of stay and the combined endpoint ‘death/new nursing home admissions’ after 12 months.

All statistical analyses were performed using IBM SPSS Statistics version 20.

### Ethical considerations

The study was undertaken in accordance with the Declaration of Helsinki. It was approved by the Regional Committee for Ethics in Medical Research in Norway and the Scotland A Regional Ethics Committee. At both sites, informed consent was obtained from the patients or from proxy decision-makers if patients did not have capacity to consent.

## Results

**Fig. 1:**
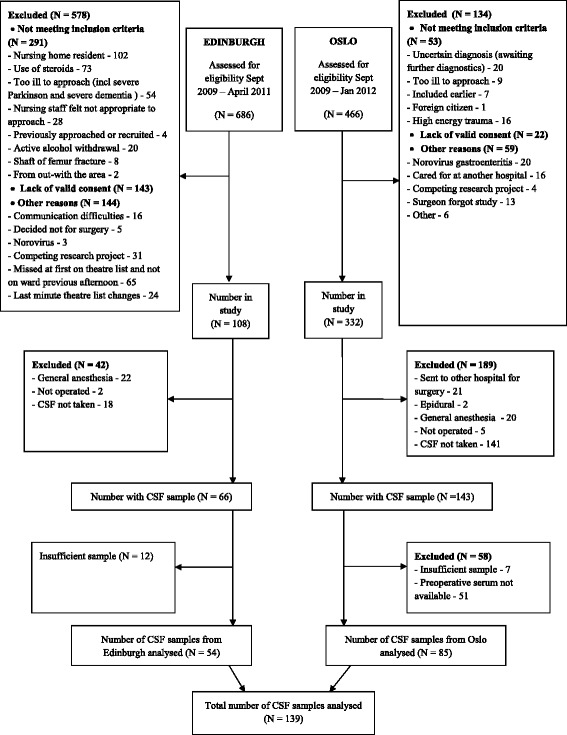
Flow chart

Paired CSF and serum samples were available from 139 hip fracture patients (Oslo *n* = 85; Edinburgh *n* = 54, Fig. 1). Compared to patients recruited in Edinburgh, fewer patients in Oslo were independent in ADL and more had pre-fracture cognitive impairment. More patients in Oslo were diagnosed with delirium (Table 1). Neopterin was detectable in all samples. No patients had a diagnosis of HIV.

**Table 1:** Baseline characteristics of hip fracture patients. Patients are stratified by centre and delirium status

	Centre			Delirium status during hospital stay^h^		
	Oslo (*n* = 85)	Edinburgh (*n* = 54)	*p* value	Delirium (*n* = 64)	No delirium (*n* = 73)	*p* value
Age, median (range)	84 (60–96)	83 (61–95)	0.18	85 (66–96)	82 (60–96)	0.02
Male (%)	19 (22)	17 (32)	0.23	19 (30)	17 (23)	0.40
IQCODE, median (IQR)^a^	3.66 (3.06–4.18)	3.00 (3.00–3.27)	<0.001	3.81 (3.08–4.80)	3.03 (3.00–3.31)	<0.001
Delirium during hospital stay (%)	43 (51)^h^	21 (39)	0.16			
Independent in ADL (%)^b^	36 (42)	49 (91)	<0.001	28 (44)	56 (77)	<0.001
Living in an institution (%)^c^	30 (35)	0	<0.001	21 (33)	7 (10)	0.001
Presence of infection and/or cancer pre-operatively (%)	11 (13)	11 (20)	0.24	12 (19)	10 (13.7)	0.42
APACHE II score, median (IQR)^d^	9 (7.3–10)	8 (6.8–10)	0.03	9 (8–10)	8 (7–10)	0.10
Charlson Comorbidity Index score, median (IQR)	1 (0–2)	1 (0–2)	0.66	1 (0–2)	1 (0–2)	0.06
Use of NSAIDs and/or ASA	23 (27)	23 (43)	0.06	22 (34)	24 (33)	0.85
Time from admission to surgery in hours, median (IQR)^e^	28.6 (19.8–42.5)	35 (23–45.5)	0.10	36.1 (23.0–46.4)	27.4 (19.8–39.5)	0.02
Length of stay, days median (IQR)	10 (7–14)	20 (13–51)	<0.001	13 (8–21.5)	11 (8–17)	0.52
Serum neopterin, median (IQR) nmol/mL^f^	28.6 (23.4–41.9)	36.1 (24.2–49.2)	0.13	37.0 (26.2–51.1)	27.1 (22.6–40.7)	0.003
CSF neopterin, median (IQR) nmol/mL^g^	25.1 (20.1–37.6)	25.9 (20.8–33.0)	0.76	29.6 (22.3–40.4)	24.7 (19.4–30.6)	0.003

*IQCODE* informant questionnaire on cognitive decline in the elderly, *IQR* interquartile range, *ADL* activities of daily living, *APACHE II* Acute Physiology and Chronic Health Evaluation II, *NSAIDs* non-steroid anti-inflammatory drugs, *ASA* acetylsalicyclic acid

^a^IQCODE was missing in 1 patient in Oslo and 4 in Edinburgh

^b^Barthel ADL ≥19 (Oslo) or Katz ≥5 (Edinburgh)

^c^Nursing home residents were excluded in Edinburgh

^d^Arterial blood gas and haematocrit omitted from formula. Missing in one patient in Oslo

^e^Waiting time for surgery was missing in three patients from Edinburgh. One patient in Oslo did not undergo surgery

^f^In six patients from Edinburgh, it was not possible to measure neopterin in serum due to an insufficient sample volume

^g^In two patients from Oslo, it was not possible to measure neopterin in CSF due to an insufficient sample volume

^h^Delirium status was uncertain in 2 patients in Oslo

### Neopterin and delirium

**Fig. 2:**
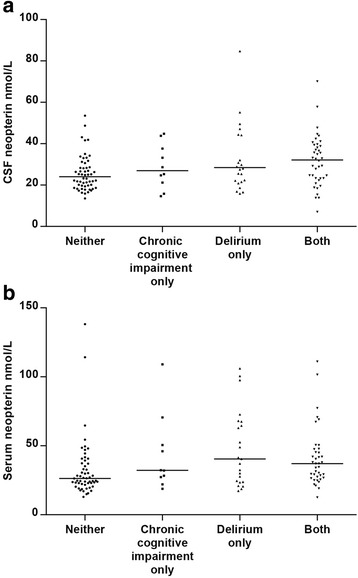
Neopterin levels in CSF (**a**) and serum (**b**) in hip fracture patients stratified regarding acute (delirium) and chronic cognitive impairment (neither: *n* = 56, chronic cognitive impairment only = 10, delirium only = 24, both = 40) *p* = 0.05 for CSF and *p* = 0.01 for serum (Kruskal-Wallis tests). The *horizontal lines* represent the median. Three patients (one with ‘neither’ and two with ‘delirium only’) had CSF neopterin above the upper margin of this figure. The Informant Questionnaire on Cognitive Decline in the Elderly (IQCODE) cutoff of 3.44 was used as an indicator of chronic cognitive impairment (missing in five patients)

Sixty-four (46 %) of the hip fracture patients developed delirium at any time. Compared to patients without delirium, delirious patients had higher levels of neopterin in CSF (median 29.6 vs 24.7 nmol/mL, *p* = 0.003) and in serum (median 37.0 vs 27.1 nmol/mL, *p* = 0.003), Table 1). Stratified analysis showed that patients with both delirium and chronic cognitive impairment had highest levels of neopterin and those with neither had lowest. A similar pattern was found in both CSF (*p* = 0.05, Fig. 2a) and in serum (*p* = 0.009, Fig. 2b).

**Fig. 3:**
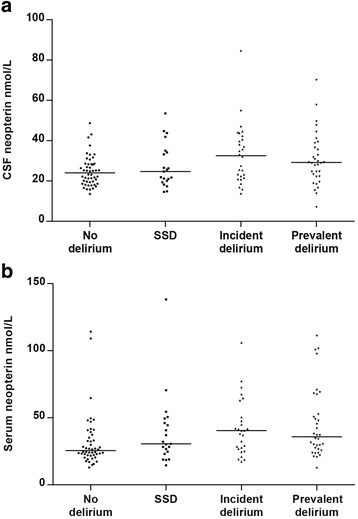
Neopterin levels in CSF (**a**) and serum (**b**) by delirium status. *p* = 0.03 for CSF and *p* = 0.001 for serum (Kruskal-Wallis tests). No delirium (*n* = 50): patients that never had delirium or SSD (subsyndromal delirium). SSD (*n* = 21): patients that had some features of delirium but never fulfilled the criteria for full delirium. Incident delirium (*n* = 28): patients that were free from delirium when sample was taken but developed delirium later. Prevalent delirium (*n* = 35): those who had delirium when the samples were taken. The *horizontal lines* represent the median. Pre-operative delirium status missing in three patients. Three patients (one with ‘no delirium’ and two with ‘prevalent delirium’) had CSF neopterin above the upper margin of this figure

In both CSF and serum neopterin was highest in patients with incident delirium (*n* = 28), followed by those with prevalent delirium (*n* = 35). The group with subsyndromal delirium (*n* = 21) had neopterin levels between the levels of patients with no signs of delirium (*n* = 50) to those with full syndromal delirium (Fig. 3).

In patients with delirium, there was a significant correlation with delirium severity and neopterin levels in the group without chronic cognitive impairment (CSF: rho = 0.42, *p* = 0.044; serum: rho = 0.44, *p* = 0.037). The same was not seen in patients with chronic cognitive impairment (CSF: rho −0.05, *p* = 0.74; serum: rho = −0.17, *p* = 0.30).

There was a near-linear relationship between delirium rates and neopterin levels in serum. For CSF neopterin levels there was a ‘cutoff’ at the 75th percentile with a steep rise in delirium rates above this ‘threshold’. In the logistic regression analysis, serum neopterin was therefore used as a continuous variable, whereas CSF neopterin was dichotomized at the 75th percentile. Neopterin remained a significant predictor of delirium in CSF (*p* = 0.02) and showed a trend in serum (*p* = 0.08) when adjusting for other risk factors in the logistic regression. The analysis identified two other risk factors for delirium: waiting time for surgery and higher IQCODE (see Table 2 for model with CSF neopterin).

**Table 2:** Logistic regression models of factors associated with delirium at any time

	Unadjusted models			Adjusted model		
	OR	95 % CI	*p* value	OR	95 % CI	*p* value
Age (years)	1.07	1.02 to 1.11	0.004			
IQCODE	4.56	2.43 to 8.52	<0.001	5.81	2.77 to 12.21	0.001
APACHE II score	1.11	0.96 to 1.27	0.15			
Independent in ADL	0.24	0.12 to 0.49	<0.001			
Time from admission to surgery, hours	1.02	1.00 to 1.05	0.046	1.04	1.01 to 1.07	0.017
Living in an institution	4.61	1.80 to 11.76	0.001			
CSF Neopterin, nmol/L^a^	3.67	1.58 to 8.47	0.002	3.31	1.24 to 8.86	0.017
Centre (reference: Oslo)	0.59	0.30 to 1.19	0.14	0.58	0.22 to 1.52	0.27

*n* = 127 in the regression analyses, some patients had to be excluded due to missing data. Variables associated with delirium in univariate analysis (*p* < 0.1) were considered for inclusion in the multivariate model, and variables were removed one by one by backwards stepping until the final model was reached. Centre (Oslo or Edinburgh) was, however, forced into the final model

*IQCODE* informant questionnaire on cognitive decline in the elderly, *APACHE II* Acute Physiology and Chronic Health Evaluation II, *ADL* activities of daily living, *CSF* cerebrospinal fluid

^a^CSF neopterin dichotomized at the 75th percentile

### Neopterin and other outcomes

There was a significant correlation between neopterin levels and length of stay in the whole study sample, both in serum (rho = 0.20, *p* = 0.025) and in CSF (rho = 0.22, *p* = 0.012). Twelve months after surgery, 32 patients had died and 13 had been newly admitted into a nursing home. When death and new nursing home admissions were combined as ‘poor outcome’, both CSF and serum neopterin levels during hospital stay were significantly higher in patients with this poor outcome compared to those without (CSF: median (IQR) 29.2 (22.8–39.6) nmol/L vs 24.9 (19.4–33.2) nmol/L, *p* = 0.02; serum: median (IQR) 37.9 (26.8–54.4) nmol/L vs 29.2 (23.1–41.7) nmol/L, *p* = 0.03).

### Subgroup analyses

There were no significant differences in neopterin levels in CSF (*p* = 0.30) or serum (*p* = 0.21) between patients who were taking (*n* = 46) or were not taking NSAIDs/ASA (*n* = 93). There were, however, trends that patients with a history of cancer and/or an ongoing infection at the time of admission (*n* = 22) had higher levels of neopterin in CSF (median (IQR) 30.3 (21.3–48.7) nmol/L vs 25.3 (19.8–33.8) nmol/L, *p* = 0.08) and serum (median (IQR) 49.4 (24.7–69.3) nmol/L vs 29.4 (23.4–41.7) nmol/L, *p* = 0.01). Accordingly, we carried out subgroup analyses excluding these patients; the association between neopterin and delirium remained strong, both in CSF (median (IQR) 29.3 (22.6–39.6) nmol/L vs 23.2 (18.6–28.5) nmol/L, *p* = 0.001) and in serum (median (IQR) 37.0 (26.8–48.9) nmol/L vs 26.4 (22.0–33.1) nmol/L, *p* < 0.001). When regression analyses were performed only with patients without cancer and/or infection, neopterin was significantly associated with delirium both in the models with serum (*p* = 0.01) and CSF (*p* = 0.01).

### Relationship between neopterin in serum and CSF

There was a significant correlation between neopterin levels in CSF and serum (rho = 0.65, *p* < 0.001). The median CSF/serum ratio was 0.81, and there was no difference in patients with (median ratio 0.81) and without delirium (median ratio 0.81, *p* = 0.62).

## Discussion

This study found novel evidence of elevated CSF levels of neopterin in delirium. This was present even when participants with active infection and malignant disease were excluded. These findings also confirm, though in a different population, those of Osse et al. of elevated serum neopterin in delirium [21]. These results suggest that there may be greater activation of the cellular immune response in delirium. They may also imply a role for oxidative stress in delirium.

The source of CSF neopterin may be from the periphery via the blood-brain barrier, but neopterin is also produced in significant quantities in the CNS, primarily by microglia [17]. The neopterin molecule itself is small, polar and stable in vivo with little cellular reuptake or metabolic degradation, being renally excreted. CSF levels of neopterin are generally half of those found in serum, and CNS production is thought to be likely if the CSF/serum ratio is greater than 1 [18, 35]. The ratio documented in this population was approximately 0.8 in all groups, suggesting that, as the ratio is greater than 0.5, there might be some in situ CNS production. Notably, Kuehne et al. have previously demonstrated that CSF neopterin is brain-derived in psychiatric patients and normal controls [35].

Elevation of neopterin in CSF in delirium points towards the potential involvement of several pathogenetic mechanisms, each of which needs to be explored in more depth by future studies. Increased oxidative stress is one plausible pathway [13]. Disruption in the energy supply required to meet the brain’s high metabolic demands likely results in acute disruption of neurotransmission, and the brain’s sensitive attentional pathways could plausibly be affected by this [5]. Activation of a cell-mediated immune response would involve activation of microglia, the brain’s resident macrophages. It is known from animal models that microglia in aged brains and those with neurodegenerative disease are ‘primed’ by these processes such that they respond more vigorously to peripheral immune stimuli [36]. This is associated with a transient decline in working memory (modelling the transient decline in cognition in delirium) and also acceleration of the chronic neurodegenerative disease process, and a parallel pathway is plausible here.

We found that patients with no underlying cognitive impairment and no delirium had the lowest levels of neopterin, and those with delirium superimposed on chronic cognitive impairment had the highest levels. The elevated levels in patients with chronic cognitive impairment is consistent with what might be expected in the presence of microglial priming, where pre-existing neurodegeneration renders microglia more likely to produce higher levels of pro-inflammatory mediators in response to acute inflammatory stimuli. In patients with delirium but no known pre-fracture dementia, this might not only reflect pre-existing milder neurodegeneration and microglial priming but may also be due to alternative processes. It may be that both delirium and dementia processes activate these systems with an additive effect observed in those with both diagnoses.

CSF neopterin has been shown to be elevated in CNS infections, including the acute phase of aseptic meningo-encephalitis, with normalization following clinical recovery [37]. It is also elevated in HIV, particularly with concomitant neuropsychiatric sequelae [18] including AIDS dementia [38]. It is present but not elevated in CSF in Alzheimer’s dementia [20], and reduced in Parkinson’s disease, reflecting a lack of biosynthesis and degeneration of nigrostriatal pathways [39].

We found higher CSF neopterin levels in those who had a poor outcome (death or new admission to care home) at 1 year. High neopterin levels, indicating greater immune system activation and oxidative stress, have been shown to be associated with poor outcomes in a range of conditions. High neopterin levels in cancer are associated with poorer survival, and the development of tumour-associated problems [16], they confer worse prognostic risk in HIV infection [15] and predict poor outcome in herpetic encephalitis [40].

This study has some limitations that should be acknowledged. Due to the cross-sectional design, we are unable to infer causation, although longitudinal CSF studies in delirium are practically and ethically difficult. Due to the nature of the different parent studies at the two sites, the Oslo cohort was frailer than the Edinburgh cohort. It is also difficult to rule out that there might have been some differences in diagnostic judgment between the two centres. The assessment tools for delirium were, however, similar and thorough and both centres used CAM as the final diagnostic tool, and we included study site in the logistic regression analyses to minimize bias.

## Conclusions

This study is the first to examine neopterin levels in CSF in delirium. We found higher levels in delirium. We also replicated a prior study showing higher serum neopterin in delirium. Our findings suggest potential roles for activation of cell-mediated immune responses and/or oxidative stress in the delirium process. High levels may also be useful in predicting poor outcomes. Future work should examine these results in the context of other CSF biomarkers, including markers of oxidative stress, IFN-γ and other components of the cellular immune response. It would also be useful to examine CSF and serum neopterin levels in delirium in other patient populations, contributing to the overall body of knowledge examining the role of inflammatory processes in delirium.

## Abbreviations

ADL, activities of daily living; APACHE II, The Acute Physiology and Chronic Health Evaluation II; ASA, acetylsalicyclic acid; CAM, confusion assessment method; CNS, central nervous system; CSF, cerebrospinal fluid; DRS, 98, Delirium Rating Scale Revised-98; GTP, guanosine triphosphate; HPLC, high-performance liquid chromatography; IFN-γ, interferon-γ; IQOCDE, The Informant Questionnaire on Cognitive Decline in the Elderly; MDAS, Memorial Delirium Assessment Scale; MMSE, Mini Mental Status Examination; NSAIDs, non-steroid anti-inflammatory drugs
